# The Effects of Barbell Placement on Kinematics and Muscle Activation Around the Sticking Region in Squats

**DOI:** 10.3389/fspor.2020.604177

**Published:** 2020-11-11

**Authors:** Roland van den Tillaar, Tom Roar Knutli, Stian Larsen

**Affiliations:** Department of Sports Sciences and Physical Education, Nord University, Bodø, Norway

**Keywords:** EMG, resistance exercise, powerlifting, high bar, low bar

## Abstract

The current study investigated the effects of barbell placement on kinematics and muscle activity during the sticking region of back squats. Ten healthy medium- to well-trained male powerlifters [age 26.1 ± 11.2 years, body mass 90.2 ± 18.3 kg, height 1.83 ± 0.09 m, five repetition maximum (5RM) 158 ± 29 kg] with at least 3 years of resistance-training experience were recruited. In a single session, participants performed 5RM movements using high bar and low bar squats, where absolute load, descent depth, and stance width were matched between squat conditions. The final repetition was analyzed using 3D kinematics and electromyography (EMG) around the sticking region. No differences in barbell and joint kinematics were observed in any phase, between both barbell modalities. Increased muscle activity in the rectus femoris, vastus medialis, and lower part of the erector spinae with the high bar, when compared with low bar conditions, was recorded. Furthermore, the gluteus maximus and medius had increased muscle activity over the three regions (pre-sticking > sticking > post-sticking), while the erector spinae, soleus, vastus lateralis, and rectus femoris experienced decreased muscle activity during the ascending phase. When depth and stance width were matched, the low bar technique was associated with lower erector spinae and quadriceps activity than the high bar technique. Thus, when the goal is to maximally activate knee extensors and the external load is matched, high bar placement would appear preferable.

## Introduction

Barbell back squats are a commonly used exercise within general fitness programs, and as an event within the sport of powerlifting, they are often included in rehabilitation programs for the lower body (Kompf and Arandjelovic, 2017). Full barbell back squats are performed by flexing the hips and knees and lowering the body until the top surface of the legs at the hip joints are lower than the top of the knees, essentially sitting down, while keeping the feet flat on the ground, chest up, and spine in neutral according to International Powerlifting Federation rules (International Powerlifting Federation, 2020). After this, the lifter reverts this movement, standing upright by extending the hips, knees, and ankles (Schoenfeld, 2010; Kompf and Arandjelovic, 2017). There are two main barbell placements in the back squat: high bar and low bar. During the high bar back squat, the barbell is placed across the upper trapezius, while in the low bar back squat, the barbell is placed along the mid trapezius, using the posterior deltoids as a supportive “shelf” for the bar (Wretenberg et al., 1996).

In powerlifting contests, the low bar barbell squat is primarily used, with a few exceptions, where the high bar back squat is used. Equally, the high bar is favored by Olympic weightlifters to simulate the catch position of the Olympic weightlifting competition lifts, the clean, snatch, and jerk (Wretenberg et al., 1996). The low bar back squat is recommended when the main objective is to lift as heavy as possible (Glassbrook et al., 2019). One of the main reasons for this is shorter moment arms, and easier work conditions for hamstrings, gluteus, and adductor muscles (Glassbrook et al., 2017). Furthermore, powerlifters produce a higher peak force in the hip joint, when compared with weightlifters performing high bar squats, while weightlifters produce a higher force in the knee joint, when compared to powerlifters (Wretenberg et al., 1996). Wretenberg et al. (1996) compared powerlifters to Olympic weightlifters for the different lifts, each performing with their own barbell placement (low or high). Thereby, it is not clear if differences can be attributed solely to the different bar positions during the lifts as it was a between-subject design in which each group performed either with low (powerlifters) or high bar (weightlifters). Furthermore, only 65% of one repetition maximum (1RM) was used in that study. Glassbrook et al. (2019) performed a kinematic and kinetic analysis investigating low and high bar squats up to loads of 1RM and observed that when using low bar back squats, more load could be lifted (+6.1–6.9%). This extra load could be lifted with more forward lean, likely to engage the hip muscles more than with high bar placement. However, they did not study muscle activity or lower-body joint angles or forces, therefore omitting what is often referred to as the sticking region.

The sticking region is where most lifts fail during training and competition (Van Den Tillaar and Ettema, 2009, 2010). It records the highest to the lowest velocity, after which barbell velocity increases again (Madsen and Mclaughlin, 1984; Van Den Tillaar and Ettema, 2009). For bench pressing, several studies (Elliott et al., 1989; Van Den Tillaar and Ettema, 2009, 2010) have shown that the sticking region occurs due to a poor biomechanical region, which means less force can be produced. For squats, also a few studies have investigated the sticking region (Van Den Tillaar et al., 2014b; Van Den Tillaar, 2015, 2019; Saeterbakken et al., 2016). In these studies, low descent velocity has a negative effect on the sticking region (Van Den Tillaar, 2019). Furthermore, the studies suggest that timing and activity between knee extensors (lateral vastus, rectus femoris) and the gluteus maximus are responsible for the sticking region, together with large joint moment arms in this region (Van Den Tillaar, 2015). Van Den Tillaar (2015) found that the quadriceps muscles decreased activity during the lift, while gluteus increased activation during the sticking region. In addition, timing of the peak and minimal angular velocities of the hip extension, knee extension, and plantar flexion movements during the ascending phase of the lift were associated with the events around the sticking region, indicating that coordination between muscles and joint movements are of main importance to surpass the sticking region.

To the best of our knowledge, no studies have yet investigated the effects of high and low bar placement on the sticking region (Van Den Tillaar and Ettema, 2009). By investigating kinematics and muscle activation around the sticking region of both squat techniques, the current study can provide information on the sticking region. Furthermore, it will provide information on which muscles help lifters through the sticking region and provide explanatory insights into those who either train the back squat, compete in powerlifting competition, or prescribe the back squat to athletes/patients as part of the training/rehabilitation program. Therefore, the aim of the study was to investigate whether bar position (low bar and high bar) affects the back squat sticking region within the sticking region performed with the same absolute external load on kinematics, muscle activity, and joint angles. The hypothesis was that the sticking region is shorter in both distance and duration during the low bar technique, when compared with the high bar technique, due to shorter lever arms especially of the trunk (Glassbrook et al., 2017, 2019) and a smoother transition from rectus femoris use to muscle activity of the posterior chain (hamstrings, glutes, and erector spinae) (Van Den Tillaar, 2015).

## Methods

### Experimental Approach to the Problem

To investigate the effects of barbell placement on barbell and joint kinematics and muscle activation around the sticking region, a repeated-measures design was used. Each participant repeated 5RM squats, with low and high bar placements in a counterbalancing order.

### Participants

Ten healthy trained male competitive powerlifters (age 26.1 ± 11.2 years, body mass 90.2 ± 18.3 kg, height 1.83 ± 0.09 m) with at least 3 years of resistance training in squats were recruited. Participants were familiar with both squat techniques as they use both techniques in their training. Inclusion criteria included that they be capable of lifting 1.5 times their own body mass in 1RM squat (femur parallel to the floor), with good technique determined by the test leader who is an experienced powerlifting trainer. Participants had no injuries that could impact their performance. No participants performed leg resistance training 24 h before testing. Participants were informed verbally and by writing of possible study risks. All provided written consent before inclusion. The study complied with current ethical regulations for research and was approved by the National Center for Research Data, in accordance with the latest revision of the Declaration of Helsinki.

### Procedures

A 5RM test was used to investigate kinematics and muscle patterns around the sticking region, during a high bar and low bar squat. 5RM repetition range is a typical training load used to increase maximal strength (Baechle and Earle, 2000), and participants were familiar with five repetitions in both techniques.

One familiarization test was conducted 2 weeks before the main study. In this test, an approximate 5RM load was predicted by participants. Approximately 90% of their estimated 5RM was used during this test, where they squatted with both techniques in a random order. Participants used their preferred stance width in which they felt comfortable to perform both types of lifts, and this position was then controlled (same stance width in both conditions) and used for subsequent attempts to prevent differences in width stances between the two conditions from influencing the moment arms around the different joints. All participants wore weightlifting shoes during all attempts. A minimum depth requirement was that the hip joint had to be lower than the knee joint in accordance with the IPF (International Powerlifting Federation, 2020). The depth was measured and marked with a horizontal rubber band.

On test day, participants started at 95% of estimated 5RM and added 2.50–7.50 kg until their real 5RM was attained. Then, they shifted to the other technique, and repeated this. Participants had one to three attempts and a 4- to 5-min pause between attempts and conditions. In a random counterbalanced order, half of the participants started with the low bar, while the other half started with the high bar technique. Participants performed a specific warm-up protocol before testing and at the familiarization test, consisting of five sets with different loads based on their 5RM: eight repetitions with a 20-kg barbell, six repetitions with 35%, five repetitions with 55%, three repetitions with 70%, and two repetitions at 90% of 5RM in squatting.

Testing was performed using an Olympic barbell (2.8 cm diameter, 1.92 m length) (Eleiko International, Halmstad, Sweden) in a weightlifting rack (Eleiko International, Halmstad, Sweden). Participants performed repetitions in a self-paced tempo environment, from full knee extension until the hamstring touched the rubber band, and returned to the starting position.

### Measurements

**Figure 1:**
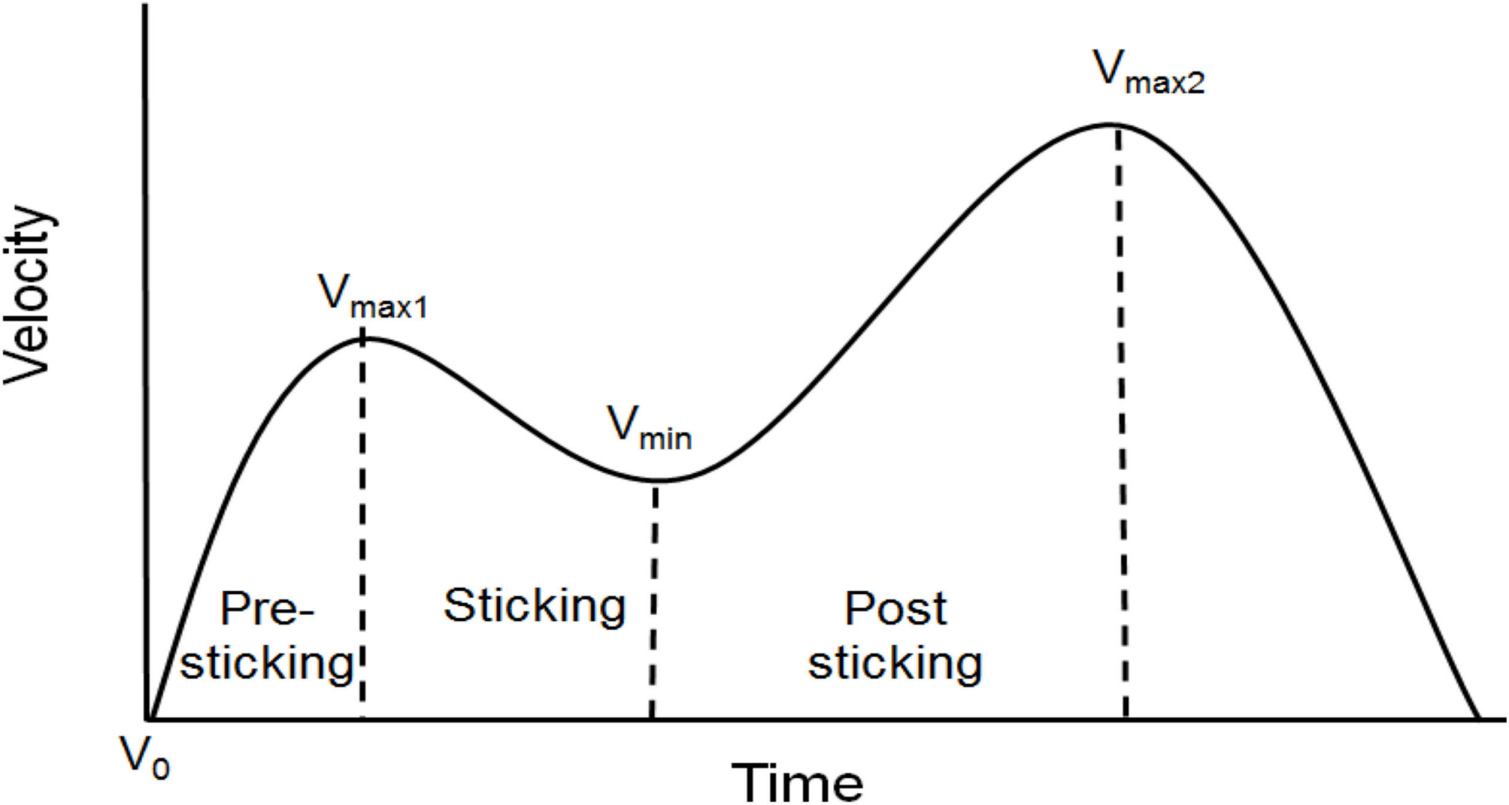
Typical barbell velocity development during a squat with a sticking region, with different events, e.g., lowest barbell height (*V*_0_), first maximal velocity (*V*_max1_), lowest velocity (*V*_min_), and second maximal velocity (*V*_max2_), and different regions.

A linear encoder (ET-Enc-02, Ergotest Technology AS, Langesund, Norway) attached to the inside of the barbell measured barbell distance and velocity over time, with a resolution of 0.019 mm and a 200-Hz sampling rate. Barbell velocity was calculated using a five-point differential filter, using Musclelab v10.190 software (Ergotest Technology AS, Langesund, Norway). From the last repetition of each condition, the vertical displacement and velocity of the following events—lowest barbell point (*V*_0_), first maximal barbell velocity (*V*_max1_), the lowest barbell velocity (*V*_min_), and the second maximal barbell velocity (*V*_max2_) of the concentric phase (Figure 1)—were measured, along with the timing of these events. The vertical displacement was measured in relation to the lowest point of the barbell (zero distance).

Wireless electromyography (EMG) was recorded with a sampling frequency of 1,000 Hz using a Musclelab 6000 system and analyzed by Musclelab v10.190 software (Ergotest Technology AS, Langesund, Norway). EMG activity was measured for 11 muscles: (1) lower and (2) upper part of the erector spinae, (3) gluteus maximus, (4) gluteus medius, (5) vastus lateralis, (6) vastus medialis, (7) semitendinosus, (8) rectus femoris, (9) biceps femoris, (10) soleus, and (11) gastrocnemius. Before testing, the skin was shaved, abraded, and washed with alcohol before gel-coated, self-adhesive electrodes were placed (Dri-Stick Silver circular sEMG Electrodes AE-131, NeuroDyne Medical, USA). The electrodes (11 mm contact diameter and 2 cm center-to-center distance) were placed on the dominant leg, along the presumed direction of the underlying muscle fiber, according to the recommendations by SENIAM or similar studies (Hermens et al., 2000; Van Den Tillaar and Saeterbakken, 2014; Van Den Tillaar et al., 2014a). EMG signals were converted to root mean square (RMS) signals using a hardware circuit network (frequency response 20–500 kHz, averaging constant 100 ms, total error ± 0.5%). To compare muscle activity around the sticking region under both conditions, three regions were assigned, and RMS EMG was calculated for each region (Figure 1), for the last repetition. The first region from the lowest barbell point (*V*_0_) to the first maximal barbell velocity (*V*_max1_) event was called pre-sticking. The second region from the first maximal barbell velocity (*V*_max1_) to the lowest barbell velocity (*V*_min_) event was called the sticking region, while the last region, post-sticking, was from the lowest barbell velocity (*V*_min_) to the second maximal barbell velocity (*V*_max2_).

**Figure 2:**
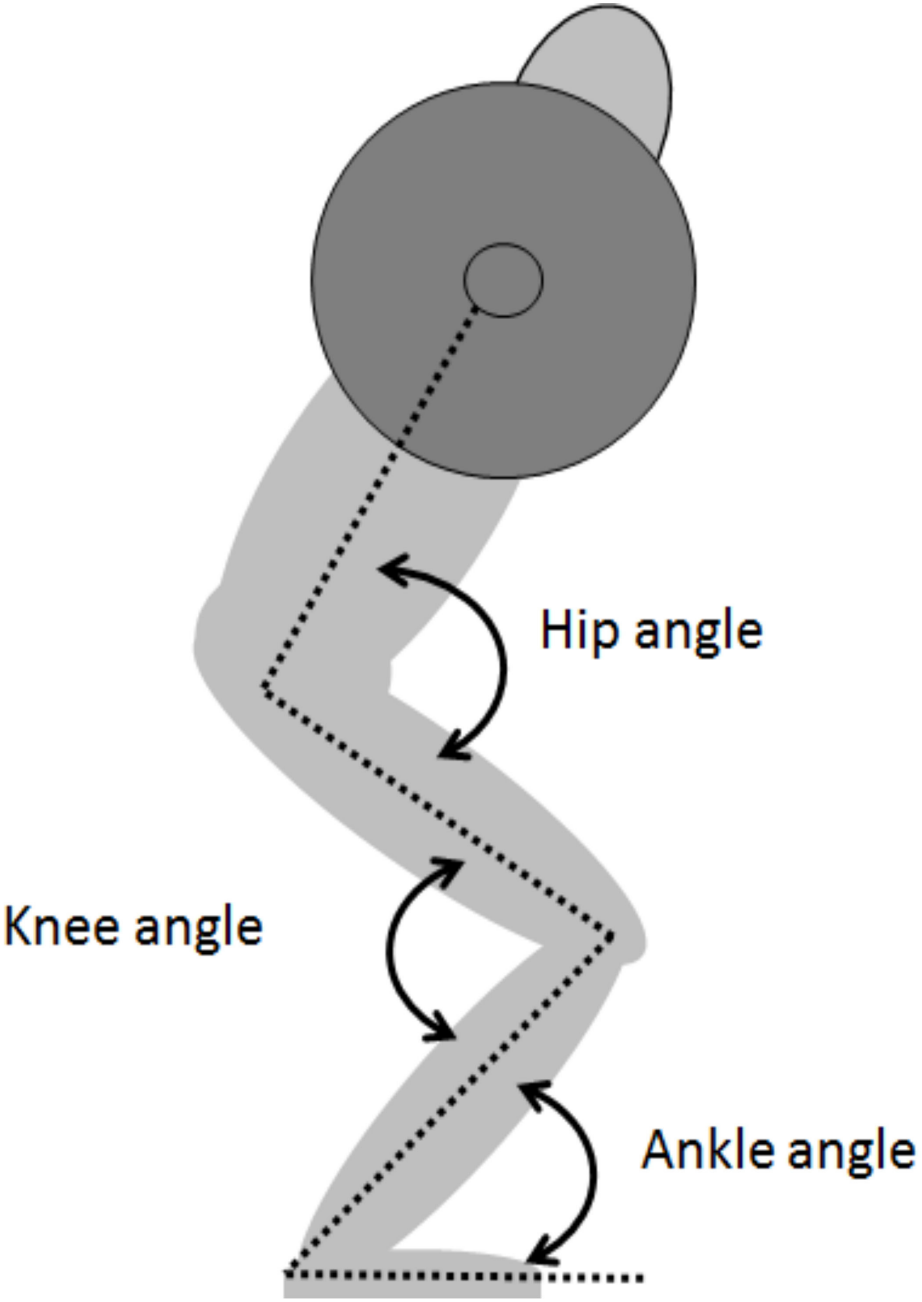
The different joint angles during squats.

A three-dimensional (3D) motion capture system (Qualysis, Gothenburg, Sweden), with eight cameras sampling at a frequency of 500 Hz, was used to track reflective markers, creating a 3D positional measurement. The 3D motion capture system was synchronized with the linear encoder and EMG recordings, using the Musclelab 6000 system (Ergotest Technology AS, Langesund, Norway). The markers were placed, one on each side of the body, on the lateral tip of the acromion, the iliac crest, greater trochanter, the lateral and medial condyle of the knee, the lateral and medial malleolus, and the distal ends of the first and fifth metatarsals. Two markers were also placed on the middle of the barbell between the hands and shoulders 80 cm apart, to track barbell displacement. Segments of the feet, lower and upper leg, pelvis, and trunk were made in Visual 3D v5 software (C-Motion, Germantown, MD, USA). Barbell position and velocity, joint angles of hip extension, knee extension, and plantar flexion were calculated using Visual 3D software. Joint angles were estimates of the anatomical angles calculated from lines formed between the center of reflective markers. The joint angles of hip extension/flexion, knee extension/flexion, and ankle plantar/dorsal flexion (Figure 2) at the fifth repetition and condition were recorded and used for further analysis at *V*_0_, *V*_max1_, *V*_min_, and *V*_max2_.

### Statistical Analysis

To assess differences in barbell and joint kinematics during events, between low and high barbell squat conditions, a one-way (low and high barbell) analysis of variance (ANOVA), with repeated measures was used. To evaluate differences in EMG activity between different conditions and regions, a 2 (conditions) × 3 (regions) ANOVA with repeated measures was performed for each muscle. If significant differences were found, a Holm–Bonferroni *post-hoc* test was performed. In cases where the sphericity assumption was violated, a Greenhouse–Geisser adjustment for *p*-values was reported. The level of significance was set at *p* ≤ 0.05. For statistical analysis, SPSS version 25.0 (SPSS, Inc., Chicago, IL) was used. All results are presented as the mean ± standard deviation (SD) and effect sizes were calculated using η^2^ (Eta squared), where 0.01 < η^2^ < 0.06 constituted a small effect, 0.06 < η^2^ <0.14 denoted a medium effect, and η^2^ > 0.14 indicated large effect (Cohen, 1988).

## Results

**Figure 3:**
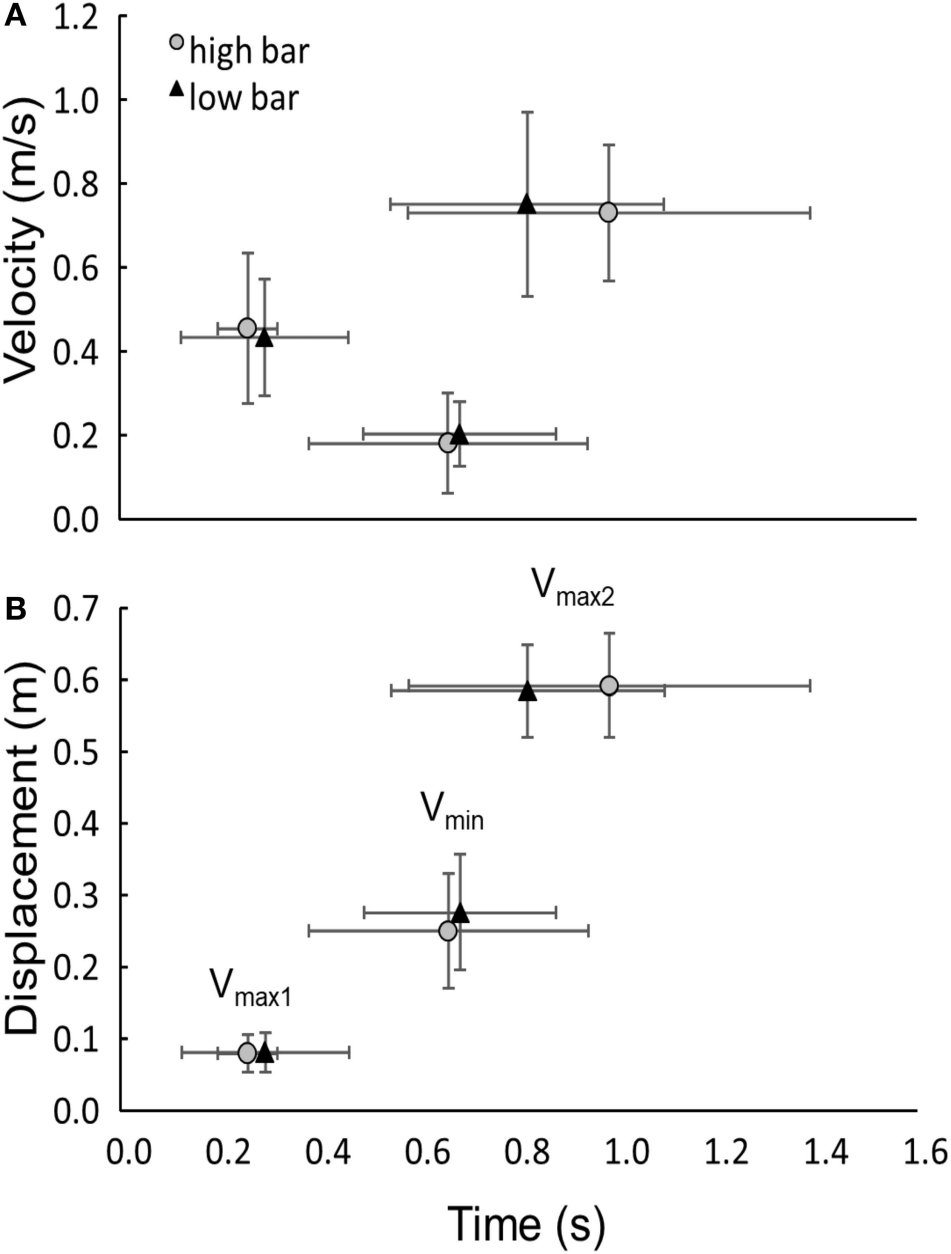
Mean (SD) **(A)** velocity and **(B)** displacement of different events and their timings, for low and high bar squats.

The average load successfully lifted by participants at 5RM was 158 ± 29 kg. No significant differences in peak velocities, distances, and timing were observed between the two barbell placements, at the different events around the sticking region (*p* ≥ 0.18, Figure 3). The sticking region started in both conditions at 12.2 ± 3.7% (*V*_max1_) and ended at 39.7 ± 9.7% (*V*_min_) of the total upwards barbell displacement. Furthermore, no significant differences were observed in ankle, knee, and hip flexion angles at any events, between high and low barbell placements (*F* ≤ 3.0, *p* ≥ 0.116, η^2^ ≤ 0.25, Table 1).

**Table 1:** Mean (±SD) of joint angles at lowest barbell point (V_0_), first maximal barbell velocity (V_max1_), minimal barbell velocity (V_min_), and second maximal barbell velocity (V_max2_) during the high bar and the low bar squat.

**Variable**	**V_**0**_**	**V_**max1**_**	**V_**min**_**	***V*_**max2**_**
**High bar squat**				
Ankle flexion (°)	70 ± 12	74 ± 11	82 ± 12	87 ± 11
Knee flexion (°)	60 ± 10	73 ± 11	108 ± 11	135 ± 7
Hip extension (°)	65 ± 21	73 ± 22	103 ± 21	128 ± 11
**Low bar squat**				
Ankle flexion (°)	72 ± 13	76 ± 13	86 ± 13	92 ± 11
Knee flexion (°)	62 ± 12	75 ± 13	114 ± 17	141 ± 11
Hip flexion (°)	62 ± 28	69 ± 30	101 ± 30	133 ± 24

A significant effect of barbell placement was observed for the rectus femoris, vastus medialis, and lower part of the erector spinae (*F* ≥ 5.6, *p* ≤ 0.045, η^2^ ≥ 0.41), with higher muscle activity in high bar conditions, when compared with low bar. Furthermore, an effect of region was observed for the gluteus maximus and medius, all three quadriceps, soleus, and both parts of the erector spinae (*F* ≥ 4.4, *p* ≤ 0.030, η^2^ ≥ 0.35), with no significant placement^*^region interaction effects (*F* ≤ 3.1, *p* ≥ 0.115, η^2^ ≤ 0.29, Table 2). *Post-hoc* comparisons revealed that the gluteus medius increased activity at each region in both barbell placements, while for the vastus lateralis and rectus femoris, the opposite occurred. The medial vastus and lower part of the erector spinae significantly decreased activity from the sticking to the post-sticking region, while the soleus decreased activity from the pre- to the sticking region. Gluteus maximus activity only increased from the pre-sticking to the sticking region (Table 2).

**Table 2:** Mean (±SEM) of muscle activation in the pre-sticking, sticking, and post-sticking region of the 11 different muscles.

	**Pre-sticking region**		**Sticking region**		**Post-sticking region**	
**Muscle (μv)**	**High bar**	**Low bar**	**High bar**	**Low bar**	**High bar**	**Low bar**
Gluteus max	33 ± 5	34 ± 5^*^	93 ± 15	93 ± 15	85 ± 10	87 ± 9
Gluteus med	30 ± 4^*^	31 ± 4^*^	83 ± 13^*^	86 ± 15^*^	115 ± 16^*^	110 ± 14^*^
Biceps femoris	80 ± 8	105 ± 21	99 ± 19	116 ± 22	136 ± 33	136 ± 27
Semitendinosis	81 ± 17	51 ± 7	102 ± 21	97 ± 16	113 ± 20	111 ± 20
Vastus lateralis	378 ± 87^*^	391 ± 90^*^	331 ± 82^*^	335 ± 77^*^	257 ± 70^*^	226 ± 51^*^
Rectus femoris^†^	310 ± 61^*^	252 ± 60^*^	246 ± 64^*^	212 ± 57^*^	119 ± 47^*^	85 ± 54^*^
Vastus medialis^†^	381 ± 89	331 ± 79	361 ± 74	315 ± 66	292 ± 46	229 ± 45^*^
Soleus	238 ± 36^‡^	231 ± 43^*^	164 ± 37	154 ± 30	124 ± 23	133 ± 24
Gastrocnemius	156 ± 61	103 ± 35	67 ± 10	52 ± 7	72 ± 22	52 ± 10
Lower erector spinae^†^	273 ± 27	202 ± 30	251 ± 20	192 ± 26	202 ± 16^*^	124 ± 22^*^
Upper erector spinae	209 ± 59	210 ± 63	202 ± 54	189 ± 54	173 ± 58	88 ± 21^*^

† Indicates a significant difference between the two conditions on a p < 0.05 level.

* Indicates a significant difference with all other regions for this condition on a p < 0.05 level.

‡ *Indicates a significant difference with all post-sticking region for this condition on a p < 0.05 level*.

## Discussion

The aim of the study was to investigate differences around the sticking region between low bar and high bar barbell back squats for barbell kinematics, muscle activity, and joint angles. The current study observed no differences in barbell and joint kinematics around the sticking region between both placements. However, increased muscle activity of the rectus femoris, vastus medialis, and lower part of the erector spinae was observed during high bar conditions, when compared with low bar. Furthermore, muscle activity of the gluteus maximus and medius increased over the three regions, while erector spinae, soleus, vastus lateralis, and rectus femoris demonstrated an opposing pattern during lifts.

No differences in barbell and joint kinematics were found around the sticking region between both barbell placements, which contradicted the experimental hypothesis and data from Glassbrook et al. (2019). The lack of differences in barbell and joint kinematics between squat variations was explained because the weight, depth, and stance width were matched between squat variations. Glassbrook et al. (2019), in a subgroup of recreationally trained athletes, recorded significant differences of 5° between low and high bar placement in the hip (high bar: ≈ 66° vs. low bar: ≈ 61°) and knee joint angles (high bar: ≈ 64° vs. low bar: ≈ 68°) at the deepest point. We recorded only differences of 2–3° (±10°) with more hip flexion and less knee flexion in the low bar placement, when compared with the high bar placement. This discrepancy was probably the result of stance width differences, which were controlled in this study, but not in Glassbrook et al. (2019). This may explain why powerlifters have even greater forward trunk lean of 10° (smaller hip joint angle) and less knee flexion (≈7°) during low bar lifts, when compared with Olympic lifters with high bar loads at the same percentage of lifting loads. A wider stance, probably used in low bar conditions, can cause lower moment arms of the barbell in comparison with the center of pressure (COP), as indicated by Glassbrook et al. (2019), which influences knee and hip joint angles (Swinton et al., 2012). However, the joint angles during the low bar squats were similar to those of regional and international leveled powerlifters from earlier low bar squat studies (Hales et al., 2009; Swinton et al., 2012; Glassbrook et al., 2019), indicating that the kinematics in our study are comparable with regional to international leveled powerlifters. Additionally, in the systematic review by Glassbrook et al. (2017), studies reporting joint kinematics in high bar variations often reported that participants squatted deeper when compared with studies reporting kinematics from a low bar variation. This could generate greater knee and ankle flexion angles at maximal depth in the high bar squat. However, in our study, depth was limited in accordance with International Powerlifting Federation requirements (International Powerlifting Federation, 2020) for approved squats, for both squat conditions, which perhaps explain the absence of differences in joint kinematics together with standardized stance width.

Although no differences in joint angles were observed between high and low bar placements, the placement had a different effect on muscle activation. Increased muscle activity of the rectus femoris, vastus medialis, and lower part of the erector spinae were recorded under high bar conditions, when compared with low bar. To our knowledge, this is the first study to directly compare muscle activities between high bar and low bar squat conditions in the same participants. Wretenberg et al. (1996) also compared muscle activity between low bar and high bar squats and observed the opposite effect, i.e., higher muscle activity for rectus femoris in low bar squats, compared with high bar squats. However, these authors compared powerlifters who squatted with the low bar technique with Olympic weightlifters who squatted with the high bar technique, and each participant could choose their preferred stance width. These factors changed the moment arms around the different joints and therefore the activity of the different muscles around these joints. Thanks to these methodological differences, it is not possible to directly compare findings between studies. However, an explanation for higher muscle activity in this study, in the lower part of the erector spinae, rectus femoris, and vastus medialis in the high bar variation, could be that higher barbell placement on the shoulder causes a larger moment arm on the hip joint, since the trunk lean is approximately the same between the high and low bar squats in the present study. The lower part of the erector spinae must be more active to resist spinal flexion and to maintain an upright trunk (Toutoungi et al., 2000). In addition, placing barbells higher on the shoulders will probably result in small individual joint angle adjustments to the ankle, knee, and hip, causing the participant to lean backwards to balance the external load above the COP. The COP moves posteriorly under the feet, due to the more proximal and anterior load to avoid a moment between this and COP. Due to this small backward displacement, the vastus medialis and rectus femoris become more active. However, no inverse dynamics could be performed showing this COP change projection due to load placement. Therefore, in future studies, kinetic analyses with inverse dynamics should be performed to investigate this.

Besides differences in muscle activities between both barbell placements, muscle activity also changed over the different regions. Activity in the glutei muscles increased from the pre- to post-sticking region, while the erector spinae, soleus, vastus lateralis, and rectus femoris decreased in muscle activity during lifts in both squat conditions, in agreement with Van den Tillaar and colleagues (Van Den Tillaar et al., 2014a; Van Den Tillaar, 2015). Van Den Tillaar (2015) observed, in the last repetition of 6RM squats, a pattern of muscular activity development comparable to that observed in this study. This was perhaps not surprising since barbell and joint kinematics were comparable in both studies. The first peak velocity (*V*_max1_) coincided with the timing of the first peak angular velocity of the knee extension and plantar flexion, while the *V*_min_ event occurred with the minimal plantar flexion and knee extension angular velocity (Van Den Tillaar et al., 2014a; Van Den Tillaar, 2015). The second peak velocity of the barbell coincided with the second peak of the plantar flexion and knee extension angular velocity and the peak hip extension velocity (Van Den Tillaar et al., 2014b; Van Den Tillaar, 2015). Since the ascending movement of the barbell started with plantar flexion and knee extension, this had to be performed by the vastus lateralis, rectus femoris, and soleus (Table 2). These muscle activities decreased around the occurrence of *V*_min_ of the barbell. The glutei muscles exhibited less activity at the start of the ascending phase (Table 2), probably due to large gluteus muscle length, and a large moment arm caused by the barbell load at this hip angle that gives mechanical disadvantages such that the capacity to exert force was reduced (Roberton et al., 2008). Plantar flexion and knee extension in the pre-sticking region changed gluteus muscle length and the moment around the hip joint, thereby facilitating more use of the gluteus during the rest of the lift (Table 2).

Our findings provide further evidence that supports Van Den Tillaar (2015), who suggested that increased gluteus muscle activity did not occur fast enough to compensate for lost muscle activity of the quadriceps in the sticking region. Therefore, a shift in muscle activity could partially explain the occurrence of the sticking region. In the current study, the gluteus medius and erector spinae were measured, but were not measured in previous studies at the sticking region (Van Den Tillaar et al., 2014b; Van Den Tillaar, 2015). Thus, it appears that the gluteus medius contributes much later to the ascending phase of the lift, while erector spinae activity decreases (Yavuz and Erdag, 2017), especially in the post-sticking region. It is accepted that squats require isometric activity from supporting muscles to stay upright, and to facilitate postural trunk stabilization (Schoenfeld, 2010). However, the decrease in erector spinae activity during the regions is probably due to large moment arms in the pre-sticking region for the erector spinae muscles, which decreases during the ascending phase. Roberton et al. (2008) observed large peak moments for the hip at the beginning of the ascending phase, when performing back squats at 80% of 1RM, which was synonymous with *V*_0_, and the start of the pre-sticking region. To enhance a proper squat technique, a rigid midsection (trunk, spine, and core) is essential to eliminate unnecessary planar motion, thus ensuring a stable trunk during the ascent. Since there is a lumbar–pelvis relationship, and the spinal angle increases when the hips are flexed (Schoenfeld, 2010), we suggest that activation of the erector spinae muscles is especially important in the pre-sticking region to withstand peak hip joint moment and therefore enhance a rigid and stable spine through the ascent. The current study also has some limitations. Firstly, the absolute load between the placements was investigated and not the relative loads. A different relative load can influence muscle activity levels and kinematics and thereby the occurrence of the sticking region. Secondly, depth was artificially controlled and the participants all wore weightlifting shoes, which could influence the findings. By wearing weightlifting shoes, it elicits changes to a participant's squat movement as it may allow a participant to achieve a greater squat depth while promoting an upright posture, especially participants who are limited in their ankle dorsiflexion (Legg et al., 2017). Another limitation of the present study is that only 10 medium- to well-trained male powerlifters were included, which perhaps is a low number of participants. This could make it difficult to generalize the findings, especially to other populations. Furthermore, no kinetics and inverse dynamics were conducted due to a lack of equipment (i.e., a 3D force platform). Therefore, in future studies, these measurements should be recorded to support observations from this study on muscle activation and mechanical challenges (moment arms and COP shift) during the sticking region and between the low and high bar placement.

## Practical Applications

No differences in barbell and joint kinematics were observed around the sticking region between high bar and low bar back squats when absolute load, depth, and stance width were controlled. Increased muscle activation of the rectus femoris, vastus medialis, and lower part of the erector spinae was observed with high bar conditions, when compared with low bar. Therefore, if the goal is to maximally activate knee extensors and the external load, minimum depth and stance width are matched, and high bar placement appears preferable. Otherwise, it is recommended to coaches and athletes that when external load, minimum depth, and stance width are matched, a low bar approach may be an advantageous choice of squat technique, due to less stress on the erector spinae, especially at the start of the ascending phase.

## Data Availability Statement

The raw data supporting the conclusions of this article will be made available by the authors, without undue reservation.

## Ethics Statement

The studies involving human participants were reviewed and approved by National Center for Research Data. The patients/participants provided their written informed consent to participate in this study.

## Author Contributions

RT conducted the data collection, supervised the study, wrote the article, and performed partly the analysis and interpretation. TK conducted the data collection, performed the initial data analysis, interpretation, and wrote parts of the article. SL performed additional data analysis, interpretation, and also wrote parts of the article. All authors contributed to the article and approved the submitted version.

## Conflict of Interest

The authors declare that the research was conducted in the absence of any commercial or financial relationships that could be construed as a potential conflict of interest.

## References

[B1] Baechle T. R. Earle R. W. (2000). Essentials of Strength Training and Conditioning. 2nd ed. Champaign, IL: Human Kinetics.

[B2] Cohen J. (1988). Statistical Power Analysis for the Behavioral Sciences. Hillsdale, NJ: Lawrence Erlbaum Associates.

[B3] Elliott B. C. Wilson G. J. Kerr G. K. (1989). A biomechanical analysis of the sticking region in the bench press. Med. Sci. Sports Exerc. 21, 450–462. 10.1249/00005768-198908000-00018 2779404

[B4] Glassbrook D. J. Brown S. R. Helms E. R. Duncan S. Storey A. G. (2019). The high-bar and low-bar back-squats: a biomechanical analysis. J. Strength Cond. Res. 33, 1–18. 10.1519/JSC.0000000000001836 28195975

[B5] Glassbrook D. J. Helms E. R. Brown S. R. Storey A. G. (2017). A review of the biomechanicsl differences between the high-bar and low-bar back squat. J. Strength Cond. Res. 31, 2618–2634. 10.1519/JSC.0000000000002007 28570490

[B6] Hales M. E. Johnson B. F. Johnson J. T. (2009). Kinematic analysis of the powerlifting style squat and the conventional deadlift during competition: is there a cross-over effect between lifts? J. Strength Cond. Res. 23, 2574–2580. 10.1519/JSC.0b013e3181bc1d2a 19910816

[B7] Hermens H. J. Freriks B. Disselhorst-Klug C. Rau G. (2000). Development of recommendations for SEMG sensors and sensor placement procedures. J. Electromyogr. Kinesiol. 10, 361–374. 10.1016/S1050-6411(00)00027-4 11018445

[B8] International Powerlifting Federation (2020). Technical Rules Book.

[B9] Kompf J. Arandjelovic O. (2017). The sticking point in the bench press, the squat, and the deadlift: similarities and differences, and their significance for research and practice. Sports Med. 47, 631–640. 10.1007/s40279-016-0615-9 27600146 PMC5357260

[B10] Legg H. S. Glaister M. Cleather D. J. Goodwin J. E. (2017). The effect of weightlifting shoes on the kinetics and kinematics of the back squat. J. Sports Sci. 35, 508–515. 10.1080/02640414.2016.1175652 27096286

[B11] Madsen N. H. Mclaughlin T. M. (1984). Kinematic factors influencing performance and injury risk in the bench press exercise. Med. Sci. Sports Exerc. 16, 376–381. 10.1249/00005768-198408000-00010 6493018

[B12] Roberton D. Wilson J. St Pierre T. (2008). Lower extremity muscle functions during full squats. J. Appl. Biomech. 24, 333–339. 10.1123/jab.24.4.333 19075302

[B13] Saeterbakken A. H. Andersen V. Van Den Tillaar R. (2016). Comparison of kinematics and muscle activation in free-weight back squat with and without elastic bands. J. Strength Cond. Res. 30, 945–952. 10.1519/JSC.0000000000001178 26349045

[B14] Schoenfeld B. J. (2010). Squatting kinematics and kinetics and their application to exercise performance. J. Strength Cond. Res. 24, 3497–3506. 10.1519/JSC.0b013e3181bac2d7 20182386

[B15] Swinton P. A. Lloyd R. Keogh J. W. L. Agouris I. Stewart A. D. (2012). A biomechanical comparison of the traditional squat, powerlifting squat and box squat. J. Strength Cond. Res. 26, 1805–1816. 10.1519/JSC.0b013e3182577067 22505136

[B16] Toutoungi D. Lu T. Leardini A. Catani F. O'connor J. J. C. B. (2000). Cruciate ligament forces in the human knee during rehabilitation exercises. Clin. Biomech. 15, 176–187. 10.1016/S0268-0033(99)00063-7 10656979

[B17] Van Den Tillaar R. (2015). Kinematics and muscle activation around the sticking region in free-weight barbell back squats. Kinesiol. Slov. 21, 15–25.

[B18] Van Den Tillaar R. (2019). Effect of descent velocity upon muscle activation and performance in two-legged free weight back squats. Sports 7:15. 10.3390/sports7010015 30621028 PMC6359524

[B19] Van Den Tillaar R. Andersen V. Saeterbakken A. (2014a). Comparison of muscle activation and performance during 6 RM, two legged free-weight squats. Kinesiol Slov. 20, 5–16.30621028

[B20] Van Den Tillaar R. Andersen V. Saeterbakken A. H. (2014b). The existence of a sticking region in free weight squats. J. Hum. Kinet. 42, 63–71. 10.2478/hukin-2014-0061 25414740 PMC4234771

[B21] Van Den Tillaar R. Ettema G. (2009). A comparison of successful and unsuccessful attempts in maximal bench pressing. Med. Sci. Sports Exerc. 41, 2056–2063. 10.1249/MSS.0b013e3181a8c360 19812510

[B22] Van Den Tillaar R. Ettema G. (2010). The sticking period in a maximum bench press. J. Sports Sci. 28, 529–535. 10.1080/02640411003628022 20373201

[B23] Van Den Tillaar R. Saeterbakken A. (2014). Effect of fatigue upon performance and electromyographic activity in 6-RM bench press. J. Hum. Kinet. 40, 57–65. 10.2478/hukin-2014-0007 25031673 PMC4096084

[B24] Wretenberg P. Feng Y. Arborelius U. P. (1996). High- and low-bar squatting techniques during weight-training. Med. Sci. Sports Exerc. 28, 218–224. 10.1097/00005768-199602000-00010 8775157

[B25] Yavuz H. U. Erdag D. (2017). Kinematic and electromyographic activity changes during back squat with submaximal and maximal loading. Appl. Bionics Biomech. 2017:9084725. 10.1155/2017/9084725 28546738 PMC5435978

